# Variability in DPA and Calcium Content in the Spores of *Clostridium* Species

**DOI:** 10.3389/fmicb.2016.01791

**Published:** 2016-11-11

**Authors:** Jan Jamroskovic, Zuzana Chromikova, Cornelia List, Barbora Bartova, Imrich Barak, Rizlan Bernier-Latmani

**Affiliations:** ^1^Swiss Federal Institute of Technology in Lausanne (EPFL)Lausanne, Switzerland; ^2^Institute of Molecular Biology, Slovak Academy of SciencesBratislava, Slovakia

**Keywords:** dipicolinic acid, sporulation, wet heat resistance, STXM, STEM-EDS, *Clostridium sensu stricto*, phylogeny, SpoVA

## Abstract

Spores of a number of clostridial species, and their resistance to thermal treatment is a major concern for the food industry. Spore resistance to wet heat is related to the level of spore hydration, which is inversely correlated with the content of calcium and dipicolinic acid (DPA) in the spore core. It is widely believed that the accumulation of DPA and calcium in the spore core is a fundamental component of the sporulation process for all endospore forming species. We have noticed heterogeneity in the heat resistance capacity and overall DPA/calcium content among the spores of several species belonging to *Clostridium sensu stricto* group: two *C. acetobutylicum* strains (DSM 792 and 1731), two *C. beijerinckii* strains (DSM 791 and NCIMB 8052), and a *C. collagenovorans* strain (DSM 3089). A *C. beijerinckii* strain (DSM 791) and a *C. acetobutylicum* strain (DSM 792) display low Ca and DPA levels. In addition, these two species, with the lowest average Ca/DPA content amongst the strains considered, also exhibit minimal heat resistance. There appears to be no correlation between the Ca/DPA content and the phylogenetic distribution of the *C. acetobutylicum* and *C. beijerinckii* species based either on the 16S rRNA or the *spo*VA gene. This finding suggests that a subset of *Clostridium sensu stricto* species produce spores with low resistance to wet heat. Additionally, analysis of individual spores using STEM-EDS and STXM revealed that DPA and calcium levels can also vary amongst individual spores in a single spore population.

## Introduction

The process of sporulation is a survival strategy for species belonging to the phylum *Firmicutes.* Bacterial endospores from this phylum, and more specifically the *Bacillales* and *Clostridiales* orders, exhibit remarkable resistance to numerous environmental insults such as heat, dessication, radiation, extremes in pH and pressure, as well as chemical oxidants. Many of clostridial spore-forming species, such as *Clostridium difficile, C. perfringens, C. botulinum, C. tetani, C. acetobutylicum*, and *C. beijerinckii*, are medically and industrially important. This group of organisms primarily uses fermentation as a metabolic strategy, but is ubiquitous in the environment and thus can be found in a wide variety of habitats, including mammalian hosts. The mechanism of sporulation is largely conserved amongst these species (Dalla Vecchia et al., 2014). However, there are large variations in the initiation of sporulation as well as in the process of spore coat formation and germination, due to differences in the environmental niches in which these bacteria thrive (Dalla Vecchia et al., 2014; Paredes-Sabja et al., 2014).

The resistance of spores to environmental attacks is attributable to the unique morphology of the spore, which is formed by several layers: the core, the cortex, the coat, the crust, and in some cases, the exosporium. These structures protect sensitive macromolecules such as DNA and crucial proteins, included in the spore core. Dipicolinic acid (DPA), with its ability to bind and chelate Ca^2+^ ions, plays a key role in the dehydration, and mineralization of the spore core. Dehydration of the spore core is the main factor underlying the resistance of spores to wet heat (Paidhungat et al., 2000).

The process of sporulation has been extensively studied for several decades for the model organism *Bacillus subtilis*. The basic morphological structure of spores is conserved in all the endospore-forming bacteria studied. This unique structure is the basis for the resistance of spores. While some of the resistance determinants of spores have been revealed and it is known that several factors contribute to the development of each type of resistance, the precise mechanism of their action remains to be uncovered (Orsburn et al., 2008). The best-studied mechanism of spore resistance is that to UV, achieved with small-acid soluble proteins (SASPs), which bind to the spore’s DNA and promote its conformational change (Setlow, 1995; Raju et al., 2006; Lee et al., 2008). Recently, it was determined that DNA was packed into crystalline nucleoids through binding by α/β type SASPs, hence protecting DNA from modification (Dittmann et al., 2015). Such protein-DNA structures allow spores to endure multiple types of environmental insults such as non-ionizing radiation, wet and dry heat, and desiccation, probably because the movement of internal molecules is restricted (Dittmann et al., 2015). In *B. subtilis*, superdormant spores were identified and were defined as spores able to withstand multiple rounds of germination conditions in the dormant state (Ghosh and Setlow, 2009; Ghosh et al., 2009).

The second most studied type of spore resistance is that to wet heat. This particular form of resistance is of major concern in the food industry because of food spoilage post sterilization. The major factor contributing to spore resistance to wet heat is water content in the spore core. In the dehydrated core, aqueous chemical reactions are inhibited, minimizing damage to biomolecules in case of exposure to thermal extremes (Setlow, 2006). It is well established that dehydration of the spore core directly correlates with its mineralization (Beaman et al., 1982). Thus, water content in the spore core is inversely proportional to the amount of DPA and Ca^2+^ (Douki et al., 2005; Setlow, 2006). DPA in the spore likely provides wet heat resistance through (a) lowering the core water content, (b) protecting proteins from denaturation from wet heat exposure, and (c) helping to stabilize DNA and to afford protection from damage (Raju et al., 2006; Setlow, 2006).

In *B. subtilis*, DPA is synthetized in the mother cell during late sporulation stages and is then taken up together with Ca^2+^ by the engulfed forespore (Tovar-Rojo et al., 2002; Li et al., 2012). The major component responsible for DPA synthesis is the SpoVF protein complex, which consists of two subunits, SpoVFA and SpoVFB (Daniel and Errington, 1993). A *B. subtilis spoVF* mutant strain forms DPA-less spores that contain a high amount of water and are less heat resistant than *spoVF* spores formed in the presence of exogenous DPA (Paidhungat et al., 2000). Exposure of such spores to wet heat results in damage to spore proteins, which is directly proportional to the time of exposure to this condition. After accumulation of defects in proteins, spores may still be able to germinate but growth into viable cells is compromised (Coleman et al., 2007). Genome analysis has revealed that although some Clostridia possess a SpoVF homolog within their genome, it is not the case for species of *Clostridium sensu stricto* (cluster I) (Onyenwoke et al., 2004). In those species, DPA synthesis could be catalyzed by the EtfA protein (electron transfer flavoprotein A), as was shown for *Clostridium perfringens* (Orsburn et al., 2010).

There is strong evidence of heterogeneity in spore populations with respect to resistance of spores to thermal treatment. A single cell analysis approach, where physiology of individual spores was monitored during the incubation at 80–90°C, revealed a variable response of spores to wet heat. The release of DPA from spores itself only takes a few minutes. However, individual spores differ in the so-called lag phase, which is the period from exposure to germination conditions until the commitment of germination, the rapid release of DPA/Ca. The lag period ranges from minutes to hours for individual spores. It is presumed that the spores with the longest lag phase are the most resistant to wet heat. However, molecular, physiological, or environmental factors underpinning this variability in spore populations are poorly understood (Coleman et al., 2007; Zhang et al., 2010, 2011).

Previous studies of resistance of spores have shown that there are large differences regarding the level of resistance to various environmental insults amongst various species (Brul et al., 2011; Maillard, 2011; Paredes-Sabja et al., 2011; Xiao et al., 2011; Setlow, 2013, 2014). In the present work, we focus on several *Clostridium* species belonging to Cluster I of genus *Clostridium* (*sensu stricto*). This cluster is morphologically and physiologically the best described group of the genus *Clostridium*, which contains over 160 species divided into multiple sub-groups according to results of 16S rRNA analysis (Gupta and Gao, 2009). Here, we examine the group of industrially important species belonging to this cluster, specifically *C. acetobutylicum* DSM1731 and DSM792, *C. beijerinckii* strains DSM791 and NCIMB 8052 and *C. collagenovorans* 3089, for their level of resistance to wet heat as well as for their DPA/Ca content. The goal of the study is to evaluate the link between heat resistance and DPA/Ca content, as well as to compare DPA/Ca levels among *Clostridium* species.

## Materials and Methods

### Bacterial Strains

**FIGURE 1:**
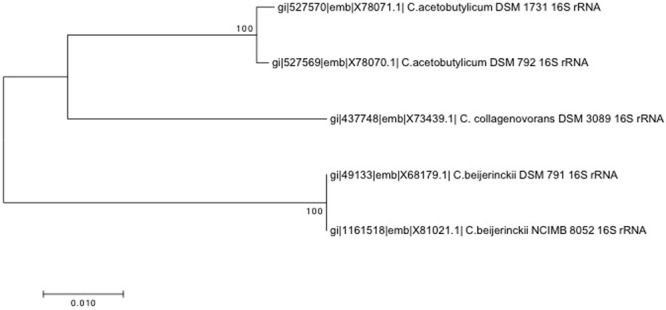
**16S rRNA-based neighbor-joining phylogenetic tree constructed using MEGA7 with the *p*-distance method and the Bootstrap method (1000 replications)**.

All the strains used in this study are listed in **Table 1**. The *Clostridium* species are closely related phylogenetically (**Figure 1**).

**Table 1:** Bacterial strains used in the study.

Strain	Source	Sporulation medium	Medium for vegetative growth
*B. subtilis* PY79	Youngman et al., 1984	DSM	LB
*C. acetobutylicum* DSM 792	Bernier-Latmani et al., 2010	FLP, CBM	CRM, 2xYTG
*C. acetobutylicum* DSM 1731	DSMZ	FLP, CBM	CRM, 2xYTG
*C. collagenovorans* DSM 3089	DSMZ	PBB	CRM, PBB
*C. beijerinckii* DSM 791	DSMZ	TGY plates, P2	CRM, TGY
*C. beijerinckii* NCIMB 8052	NCIMB	TGY plates, P2	CRM, TGY
*D. reducens* MI-1	Bernier-Latmani et al., 2010	WLP	WLP

*Medium abbreviations are explained in the text.*

*DSMZ, German Collection of Microorganisms and Cell Cultures, Braunschweig, Germany*

*NCIMB, National Collection of Industrial, Food and Marine Bacteria LTD, Aberdeen, UK.*

### Bacterial Growth and Spore Preparation

*Clostridium acetobutylicum* DSM 792 and *C. acetobutylicum* DSM 1731 were grown in anoxic conditions in clostridial reinforced media (CRM, Difco) or 2xYTG (per 1 l: Bacto-tryptone, 16 g; yeast, 10 g; sodium chloride, 5 g; glucose, 5 g). For sporulation, bacterial cultures were incubated in Francis low phosphate (FLP) basal medium (per 1 liter: CaCl_2_, 0.5 g; K_2_HPO_4_, 0.03 g; NH_4_Cl, 0.5 g; MgSO_4_, 0.2 g; FeSO_4_, 2.8 mg; glucose, 5 g; glycerol, 0.25 mL; peptone, 0.1 g; yeast extract, 0.1 g. pH was set at a value of 6.8) and cultures were incubated at 30°C (Bernier-Latmani et al., 2010) or on clostridium basal medium (CBM) agar plates (per 1 liter: MgSO_4_ × 7H_2_O, 0.25 g; MnSO_4_ × H_2_O, 9.5 mg; FeSO × 7H_2_O, 12.5 mg; para-aminobenzoic acid, 1.25 mg; biotin, 0.0025 mg; thiamin hydrochloride, 1.25 mg; casein hydrolysate, 5 g; glucose, 50 g; K_2_HPO_4_, 0.63 g; KH_2_PO_4_, 0.63 g) (O’Brien and Morris, 1971) and incubated at 37°C. Several days of incubation on agar plates resulted in sporulation. In fact, samples on CBM agar plates contained more spores than liquid cultures. The presence of spores was confirmed by light microscopy.

*Clostridium beijerinckii* DSM 791 and *C. beijerinckii* NCIMB 8052 were grown in anoxic conditions in tryptone-glucose-yeast extract (TGY) medium (per 1 liter: Bacto-tryptone, 30 g; glucose, 20 g; yeast extract, 10 g; cysteine, 1 g) or CRM (Difco) at 37°C. For spore preparation, cells were grown in TGY medium overnight and the next day 1–5% of the inoculum was transferred into P2 sporulation medium (per liter: glucose, 60 g; yeast extract, 1 g; pH was set to 6.6, resazurin, 0.5 mg; thiamine, 1 mg; biotin, 0.01 mg; K_2_HPO_4_, 0.5 g; KH_2_PO_4_, 0.5 g; CH_3_COONH_4_, 2.2 g; MgSO_4_ × 7H_2_O, 0.2 g; FeSO_4_ × 7H_2_O, 0.01 g; MnSO_4_ × 7H_2_O, 0.01 g; para-aminobenzoic acid, 1 mg) (Wang et al., 2013). The presence of spores was verified by light microscopy after several days of growth. Alternatively, a culture growing in TGY medium was spread on TGY plates and grown at 37°C in an anoxic chamber for several days, which resulted in a higher spore yield than sporulation in P2 liquid medium.

*Clostridium collagenovorans* DSM 3089 was grown in slightly modified PBB medium (for 1 liter: K_2_HPO_4_, 2.9 g; KH_2_PO_4_, 1.5 g; MgCl_2_ × 6H_2_0, 0.1 g; CaCl_2_ × 2H_2_O, 0.1 g; NaCl, 0.9 g; NH_4_Cl, 1 g; trace mineral solution, 10 ml; vitamin solution, 10 ml; resazurin [7-hydroxy-3H-phenoxazin-3-one 10-oxide], 0.2%, pH = 7.8; gelatin, 0.5%, reduced by 0.025% of Na_2_S prior to inoculation) for several days at 37°C. This medium also served as a sporulation medium. After several days of growth, the culture contained more than 80% spores without requiring specific conditions for sporulation induction.

*Desulfotomaculum reducens* M1-1 was grown anaerobically in liquid basal Widdel low-phosphate medium (WLP) for several days at 37°C (Bernier-Latmani et al., 2010). No induction of sporulation condition was required. The presence of spores was verified by optical microscopy.

*Bacillus subtilis* PY79 and *B. subtilis ΔspoVFΔsleB* were grown in DSM sporulation medium (Difco) at 37°C. After 48 h of growth, the cultures consisted of 99% spores, as was verified by optical microscopy and sporulation efficiency in case of wild-type *B. subtilis*.

In all cases, spores were collected by centrifugation, washed in cold deionized water to remove the medium and stored at 4°C. The lysis of vegetative cells was carried out using osmotic stress, low temperatures, and in case of *Clostridium* and *Desulfotomaculum* species by long-term exposure to oxygen. We assumed that after several days of storage in aerobic conditions in distilled water at 4°C, all vegetative cells were dead and only spores remained. This spore preparation was used for all subsequent experiments.

*Clostridium* reinforcement media as a rich complex medium was used in case of all analyzed strains for enumeration of spores by MPN approach, since the quick outgrowth of spores is crucial for this method.

### Scanning Transmission X-ray Microscopy (STXM) and Scanning Transmission Electron Microscopy (STEM) Analyses

Spectro-microscopic measurements and data analysis were performed as described elsewhere (Jamroskovic et al., 2014). Briefly, spore samples (2 μL) diluted to an appropriate number of spores were loaded on carbon grids (Lacey Carbon support grids, EMS) or on silicon nitride (SiNi) non-porous TEM window grids (TEM windows, West Henrietta, NY, USA) and were left to dry completely. Silicon nitride non-porous TEM window were mounted on STXM holders with conventional double-sided sticky tape. STXM analysis was conducted at the SM-beamline end station at the Canadian Light Source (Saskatoon, Canada). Carbon speciation stacks by STXM were collected through serial image collection along C K-edge energies (280–300 eV) extended to capture the calcium LII and LIII edge peaks; thus, the entire energy range considered was 280–355 eV.

Data processing was done using the axis2000 software package (Hitchcock, 1997). Stacks were iteratively aligned to convergence by cross-correlation using the Jacobsen stack analyze algorithm (Jacobsen et al., 2000) with the highest energy image as a reference. Spectra were initially evaluated and normalized to the same thickness using axis2000 before importing into Excel for direct data manipulation. Compared spectra were scaled linearly using 283 and 287 eV as endpoints for the DPA spectra and 346 and 356 eV for calcium spectra.

Scanning Transmission Electron Microscopy with energy dispersive spectroscopy (EDS) was used to obtain elemental composition maps and to perform comparative characterization of elemental content. In this study, an X-ray EDS system (Esprit/Quantax Bruker) in STEM mode in a FEI Tecnai Osiris microscope [200 kV X-FEG field emission gun, X-ray detector (Super-X) with 4 mm × 30 mm windowless SDD diodes and 0.9 sr collection angle was applied]. Quantitative EDS analysis was carried out using the Cliff–Lorimer standard-less method with thickness correction using K-series. The physical Bremsstrahlung background was calculated based on the sample composition. Some elements such as Cu contributing from the Cu grid were removed from quantification after the deconvolution procedure in the quantification process. Elemental concentrations in atomic % and net counts (signal above background) were derived from deconvoluted line intensities within a 95% confidence level. The process time and acquisition rates were adapted to get the most accurate data for specific element such as Ca, P, and Mn. The experimental spectra were collected with no pile-up artifacts. A correction for specimen drift was applied during acquisition to improve elemental mapping accuracy.

### Spore Heat Resistance Determination

To obtain the total number of spores in each spore suspension, 0.5 ml of untreated spore suspension was resuspended in 4.5 ml of appropriate anoxic medium and number of spores was determined by MPN (most probable number) method as follows. For each spore suspension, several 10-fold serial dilutions were prepared. With each increasing dilution, the likelihood of the medium being inoculated by a spore decreases. The growth of bacteria in serial dilutions was evaluated after 2–4 days of incubation at 37°C. The number of tubes with visible turbid cultures was counted and number of spores was estimated using MPN statistic tables. The number of spores in untreated samples obtained for each spore suspension was taken as 100%. Equivalent amounts of spore suspensions (0.5 ml) were treated at 85°C for 10 and 60 min. Serial dilutions of each treated sample were performed and MPN method was used to estimate the number of surviving spores, which is expressed as a percentage of the surviving spores from the untreated sample. All experiments were done in triplicate for each sample (Sutton, 2010).

### Quantification of DPA in Spores

Dipicolinic acid concentration was measured using a terbium complexation method (Rosen et al., 1997; Ammann et al., 2011). The calibration curve was prepared using three different concentrations of DPA (0.005, 0.05, and 0.5 μM) in sterile milli-Q water and TbCl_3_ at a final concentration of 30 μM. A sample containing 30 μM TbCl_3_ was used as a blank. Samples containing various concentrations of DPA without TbCl_3_ were also probed for their background photoluminescence, which was subtracted from the measurements. The photoluminescence at defined DPA concentrations was measured at least three times at an excitation/emission wavelength of 276/546 nm on a plate reader (SYNERGYMx, BioTek) and was used for calculation of the calibration curve.

A spore suspension with a known number of spores was autoclaved at 120°C for 30 min to induce the quantitative release of DPA. After autoclaving, the spore suspension was centrifuged and the supernatant was diluted several times by serial dilutions in sterile milli-Q water. TbCl_3_ was added to each dilution to a final concentration of 30 μM and the photoluminescence of terbium-DPA complexes was detected. Every sample was measured in triplicates. The concentration of DPA per spore was calculated through normalization with the number of spores obtained by the MPN method.

## Results

### Heat Resistance of Spores Belonging to Clostridial Species

Heat resistance testing of spores of *C. acetobutylicum* DSM 792 and *C. beijerinckii* DSM 791 revealed that they are rather sensitive to wet heat, with 4.6 and 3.9% survival, respectively, after the short wet heat treatment, and only 0.46 and 0.07% survival, respectively, after the prolonged wet heat treatment (**Table 2**).

**Table 2:** Table of spore survival after 10 and 60 min of wet heat treatment (80°C) expressed as the percentage of spore survival (determined by the MPN method) as compared to an untreated sample.

Strain	Percent survival after heat treatment		DPA (pg) per spore	Qualitative amount of Ca by STEM-EDS and number of spores in that category and Ca atomic % ±SD		Ca in vegetative cells atomic %	Calc. spore DPA conc. (pg/μm^3^)
	10′	60′				
***C. acetobutylicum***	4.6	0.46	1.98 ± 0.148	High	12 spores at 5.7% ±1.16	0.13	2.1
**DSM 792**				Mid	3 spores at 2.94% ±0.50		
				Low	6 spores at 0.44% ±0.14		
***C. acetobutylicum* DSM 1731**	37	37	2.6 ± 0.299	High	10 spores at 8.02% ±0.75	0.46	8.8
*C. collagenovorans* DSM 3089	100	2.5	9.25 ± 0.910	High	10 spores at 8.58% ±2.45	0.39	18.4
***C. beijerinckii* DSM 791**	3.9	0.07	0.088 ± 0.0033	Low	4 spores at 1.29% ±0.26	0.05	0.3
***C. beijerinckii* NCIMB 8052**	81.5	0.36	4.99 ± 0.377	High	10 spores at 9.04% ±0.83	0.28	4.2
				Low	2 spores at 1.14% ±0.04		
*D. reducens* MI-1	100	20	2.8 ± 0.338	High	10 spores at 7.96% ±1.47	0.20	4.2
*B. subtilis* PY79	100	100	1.35 ± 0.35	High	10 spores at 7.29% ±1.23	1.66	4.6

*Dipicolinic acid (DPA) per spore was measured by bulk terbium chloride measurement. Calcium per spore was measured by Scanning Transmission Electron Microscopy to energy dispersive spectroscopy (STEM-EDS). Solventogenic strains are indicated in bold.*

*Clostridium acetobutylicum* DSM 1731 spores showed intermediate heat resistance (37% after 10 min) and it is interesting to note that the survival is identical at 60 min of wet heat exposure. This is the only *Clostridium* species probed that exhibits this type of tailing in the survival after extended periods of exposure to wet heat. To a much lesser extent, a similar tailing was observed for *C. collagenovorans* DSM 3089. This strain is the most resistant of all the *Clostridium* strains analyzed, with 100 and 2.5% survival after the short and long wet heat treatment, respectively.

A second strain of *C. beijerinckii*, NCIMB 8052, is not defined as a type strain but has been studied extensively due to its massive solvent production. Wet heat resistance properties of this strain were shown to be distinct from those of *C. beijerinckii* DSM 791 strain, since after the short heat treatment, more than 80% of the NCIMB 8052 spores recovered. However, longer wet heat treatment resulted in significantly diminished survival, only 0.36% (**Table 2**).

The two control strains, *D. reducens* and *B. subtilis*, both show complete wet heat resistance at 10 min and either complete (100%) or significant (20%) resistance after 60 min of exposure to wet heat (**Table 2**).

### Overall DPA Levels in Spore Populations of Clostridia Cluster I

The content of DPA in *C. beijerinckii* DSM 791 was the lowest measured within the strains considered here. The average DPA content in spores of this strain was only 0.088 pg of DPA per spore (**Table 2**). In contrast, the spore population of the other *C. beijerinckii* strain (NCIMB 8052) was shown to contain a much higher amount of DPA than DSM 791 (4.4 pg of DPA per spore; **Table 2**). The two *C. acetobutylicum* strains exhibited DPA content ranging from 1.98 pg/spore (strain DSM 792) to 2.6 pg/spore (DSM 1731).

**FIGURE 2:**
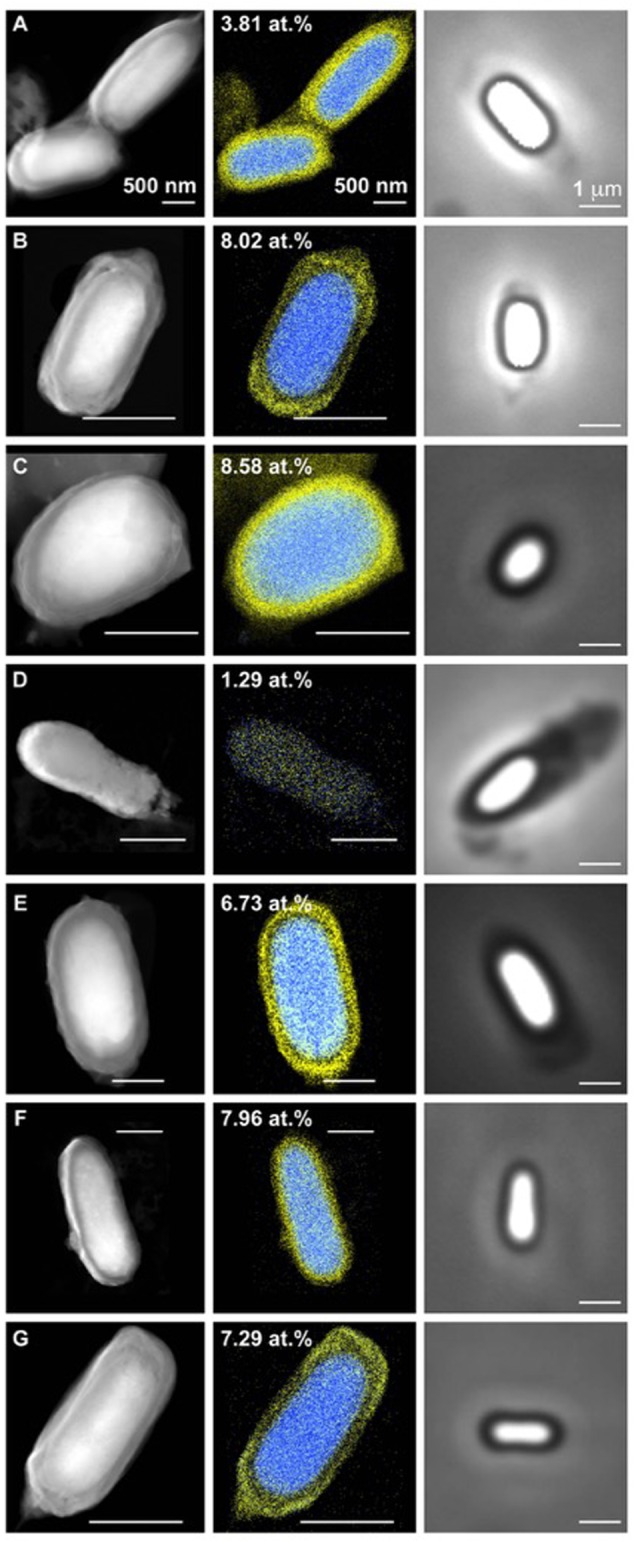
**Scanning Transmission Electron Microscopy coupled to energy dispersive spectroscopy (STEM-EDS) and bright field images of clostridial spores.(A)** *C. acetobutylicum* DSM 792 (spore width 0.9 mm), **(B)** *C. acetobutylicum* DSM 1731 (spore width 0.5 mm), **(C)** *C. collagenovorans* DSM 3089 (spore width 0.8 mm), **(D)** *C. beijerinckii* DSM 791 (spore width 0.5 mm), **(E)** *C. beijerinckii* NCIMB 8052 (spore width 1 mm), **(F)** *D. reducens* MI-I (spore width 0.75 mm), **(G)** *B. subtilis* PY79 (spore width 0.5 mm). Calcium is false colored in blue and chlorine in yellow in EDS images. Number in EDS images represents atomic percentage of calcium.

The biochemical analysis of DPA levels in spore population of *C. collagenovorans* shows very high levels of DPA per spore (9.25 pg of DPA/spore), which makes it the highest of all analyzed strains, including the control strains. Because the DPA measurement is carried out for the entire population rather than individual spores, the DPA content per spore does not represent a concentration but rather a total amount. As a result, the size of the spore is an important determinant of the concentration of DPA, which in turn, is a determinant of heat resistance. Assuming that spores on **Figure 2** represent a typical size for each strain and a cylindrical shape, we computed the volume of spores and the corresponding DPA concentration for each strain (**Table 2**).

### Spectromicroscopy Analysis of Individual Clostridial Spores

Raman spectroscopy has been previously used to measure Ca/DPA in single spores (Huang et al., 2007). Here, instead, we employed another single cell analysis approach, using a combination of two advanced spectro-microscopy techniques, STEM-EDS and STXM, which were recently optimized for bacterial spore research (Jamroskovic et al., 2014). Using these methods, we detected DPA and calcium absorption spectra directly in individual spores of each analyzed species. In STXM, the presence of DPA in the spore core appears as a characteristic double peak at 284.7 and 285.3 eV (Jamroskovic et al., 2014), while calcium levels in the spore core are characterized by the presence of a double peak at 349 and 352 eV according to the L_II_ and L_III_ edges of calcium (Jamroskovic et al., 2014).

**FIGURE 3:**
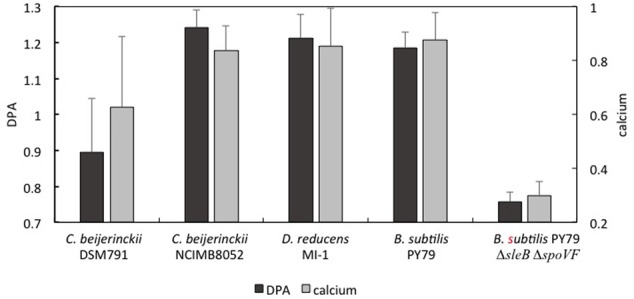
**Average values of dipicolinic acid (DPA) and calcium ions in scanning transmission x-ray microscopy (STXM) analyzed spores of several Clostridial species.** All STXM data are normalized to the same thickness of the sample. The level of DPA is expressed as a ratio of the peak at 285.35 eV to the peak at 285.05 eV (black). Calcium is expressed as an absolute value of the peak at 352 eV (gray). Primary vertical axis correspond to DPA presence, secondary vertical axis correspond to calcium level (arbitrary units).

Scanning Transmission Electron Microscopy-Energy Dispersive Spectroscopystem EDS analysis of the spores of *C. acetobutylicum* DSM 1731 and *C. collagenovorans* DSM 3089 revealed uniformly high levels of calcium in the spore core (8.02 and 8.58%, respectively; **Figure 2**). In contrast, *C. beijerinckii* DSM 791 was found to have very low calcium content both by STXM (**Figure 3**) and STEM-EDS (**Table 2**; **Figure 2**). Average values collected by STXM for all individual spores analyzed indicate relatively low level of DPA in spores (**Figure 3**).

Scanning Transmission X-ray Microscopy analysis of 13 *C. beijerinckii* NCIMB 8052 individual spores revealed a massive DPA peak at 285.3 eV comparable to the DPA peak from *B. subtilis* and *D. reducens* spores, and significantly higher than the DPA-specific peak of *C. beijerinckii* DSM 791 (**Figure 3**). The STXM signal for calcium was also substantially higher when compared to the calcium signal of *C. beijerinckii* DSM 791 (**Figure 3**).

**FIGURE 4:**
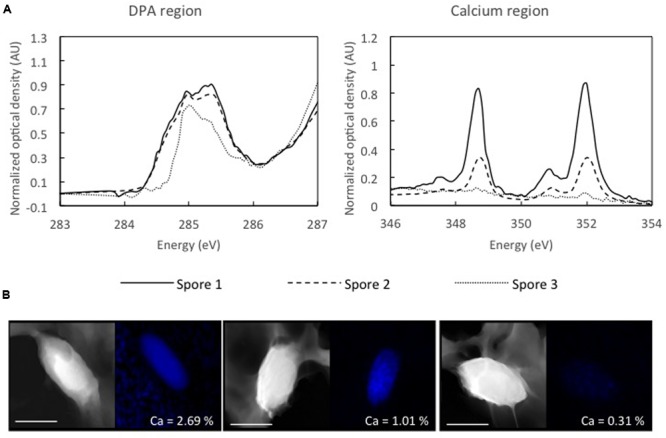
**Energy spectra for DPA and calcium regions in three different spores of *C. beijerinckii* DSM 791 obtained by STXM. (A)** Spores were chosen based on differences in calcium and DPA content, spore with low DPA and low calcium (dotted line), spore with higher DPA and higher calcium (dashed line) and spore with high DPA and high calcium levels (black line). **(B)** STEM and EDS maps of the same three spores of *C. beijerinckii* DSM 791 obtained after STXM measurement. Atomic percentage of calcium is shown for each spore. Scale bar represents 1 μm.

During analysis of *C. beijerinckii* DSM 791 by STXM, we noticed significant heterogeneity in DPA and calcium content between individual spores in the spore population of this strain (**Figure 4**). The example of this heterogeneity is highlighted in comparison of absorption spectra for DPA and calcium in three individual spores of this strain (**Figure 4**) and the STEM-EDS for the same individual spores. In one of analyzed spores (spore 3), the second peak at 285.3 eV characteristic for DPA is missing and thus this spore resembles spores which lack DPA, like *B. subtilis* mutant strain *spoFV sleB* (Jamroskovic et al., 2014). On the other hand, spores 1 and 2 show higher peaks at 284.7 and at 285.3 eV, suggesting the presence of higher amounts of DPA. The signal for calcium (double peak at 249 and 352 eV) mimics that of DPA in all three spores. The same spores were also examined by STEM/EDS microscopy after STXM analysis (**Figure 4B**). High Angle Angular Dark Field STEM microscopy (HAADF-STEM) images show the morphology of these three representative spores. The spore core, which is denser, and the cortex are clearly distinguishable in all three spores. The atomic percentage of calcium in these three spores, where spore 1 was shown to contain 2.69% Ca, spore 2 contains 1.01%, and spore 3 contains 0.31% of calcium, indicates a correlation between the level of DPA and calcium in the core and thus confirms the STXM data (**Figure 4A**).

We also examined spores of two other Firmicutes, *B. subtilis* and *D. reducens* MI-1, to ascertain whether the variability in levels of DPA and calcium in the spore population is a common feature among other endospore formers. The two species examined formed phase-bright, slightly elliptical spores (**Figure 2**) and STXM and STEM-EDS data showed the presence of high levels of Ca. STXM also indicated high levels of DPA, in contrast to bulk DPA measurements that resulted in significant but not very high values (2.8 and 1.35 μg per spore) relative to the *Clostridium* species considered (**Table 2**). This discrepancy can be attributed to the difference in spore size between the genera considered. In fact, when DPA content is normalized to volume, the content in *B. subtilis* and *D. reducens* is comparable to *C. beijerinckii* NCIMB 8052, which also forms highly wet heat resistant spores.

A major result from the STEM-EDS analysis of spores from the seven strains considered is that some strains (*C. acetobutylicum* DSM 792 and *C. beijerinckii* NCIMB 8052) exhibit great variability in calcium content while other strains (*C. acetobutylicum* DSM 1731, *C. collagenovorans, C. beijerinckii* DSM 791, *D. reducens, and B. subtilis*) show a uniform distribution of calcium amongst the spore population (**Table 2**).

As noted above, it is exceedingly difficult to obtain a clean spore-only preparation for some *Clostridium* species. However, we measured calcium content in vegetative cells of the *Clostridium* species and found it to be below that in the spores (**Table 2**). Hence, calcium content values greater than the vegetative cell values along with clear spore morphology are pre-requisites for identification of spores in STEM-EDS images.

## Discussion

Most of the studies of spore resistance strategies have been performed on *B. subtilis* spores due to the easy acquisition and large availability of existing mutants. Here, we focused on the heat resistance properties of spores of species belonging to the genus *Clostridium sensu stricto* (Cluster I).

Species analyzed in this study are very closely related phylogenetically (**Figure 1**), but they also share the same ecological niches as they are ubiquitous in soil, water, and feces; maybe with the exception of *C. collagenovorans* which was isolated from sewage sludge (Jain and Zeikus, 1989). In fact, many *C. beijerinckii* strains were considered to be *C. acetobutylicum*, until DNA fingerprint analyses and sequencing of 16S rRNA proved otherwise (Keis et al., 1995). All of these strains are anaerobic and use a fermentative type of metabolism. *C. acetobutylicum* and *C. beijerinckii* strains also share the same physiology and are able to produce solvents in the process of solventogenesis. Solventogenesis together with sporulation belong to strategies to survive extreme acidic environment, which is induced by acid-generating metabolism of *Clostridia* (Dürre, 2009). During solventogenesis, cells utilize some of the by-product acids to produce neutral solvents, and thus the pH of the environment is restored to favorable levels. In solventogenic *Clostridia*, solventogenesis and sporulation are coincident, and are both regulated by the stationary phase regulator, Spo0A (Ravagnani et al., 2000).

*C. collagenovorans* DSM 3089 is also closely related to *C. acetobutylicum* (Ludwig et al., 2009) (**Figure 1**). In addition to being acetogenic, it is also able to produce an active collagenase that can hydrolyze collagen and release peptides from animal proteins (Jain and Zeikus, 1989). In contrast to all other *Clostridium* strains considered, it is not solventogenic (Jain and Zeikus, 1988).

Our results show that despite the close relatedness of analyzed species, they exhibit large variability in calcium and DPA content as well as heat resistance, regardless of phylogeny. The *Clostridium* species considered here can be divided into several groups.

First, *C. collagenovorans* DSM 3089 behaves similarly to spore-forming organisms outside of the Clostridia. It exhibits high heat resistance, and uniformally high calcium and DPA concentrations in the core. Thus, this organism represents one end of the spectrum of spore-forming organisms. The *Clostridium* species with the next lowest wet heat resistance is *C. beijerinckii* NCIMB 8052 that shows an 81.5% resistance at 10 min of exposure and 0.36% at 60 min. The DPA concentration is comparable to that of *D. reducens* and *B. subtilis* and a large percentage of the spores characterized display a high calcium concentration (**Table 2**). However, in contrast to *C. collagenovorans*, a few spores contain low calcium concentrations (∼1 atomic %). Thus, there is sufficient intra-population variability to be identified from a relatively small sample size (12 spores).

There is a large decrease in heat resistance between the two aforementioned strains and *C. acetobutylicum* DSM 1731. Only 37% of the spores survive 10 min of heat treatment and interestingly, the same fraction survives 60 min of the same treatment. Surprisingly, the calcium and DPA concentrations are high (greater than those in *B. subtilis* and *D. reducens*). It is unclear why the wet heat resistance is much lower in the case of strain DSM 1731 than the control strains. A possible explanation for this discrepancy is that the DPA synthesis pathway is distinct for Cluster I of the *Clostridium* genus as compared to organisms outside that cluster. More specifically, the EtfA flavoprotein encoded by *etf*A, was identified as the DPA synthase in *C. perfringens*, a *Clostridium* that doesn’t possess the homolog of SpoVF DPA-synthase from *B. subtilis* (Orsburn et al., 2010). In fact, homologs of SpoVF are missing throughout the whole Cluster I of the *Clostridium* genus and it is very likely that in these species EtfA functions as the only DPA synthase. It is unclear whether this difference in pathway may also impact the timing of the production of the DPA and how it is distributed within the spore.

Finally, *C. beijerinckii* DSM 791 and *C. acetobutylicum* DSM 792 have very low wet heat resistance (3.9 and 4.6% survival after 10 min, respectively). For *C. beijerinckii* DSM 791, the low wet heat resistance is combined with low DPA and calcium concentrations (**Table 2**). Additionally, in the same strain, there is negligible heat resistance at 60 min of heat exposure, confirming the findings at 10 min. Thus, in this case, low calcium and DPA appear to correspond to low wet heat resistance. While the STEM-EDS results indicated relatively uniform Ca concentrations within the spore population, the STXM data (accompanied by the STEM-EDS data for the same spores) evidenced individual variability with a range of DPA concentrations and Ca concentrations ranging from 0.31 to 2.69 atomic %. Thus, the DPA and Ca are low but display some variability.

For *C. acetobutylicum* DSM 792, we found a wide distribution of calcium concentrations in spores (**Table 2**). A large fraction (57%) of the spores characterized exhibited an average calcium content of 5.7 atomic % while the rest contained an average of 2.94 atomic % for three spores and 0.4 atomic % for six spores. The average spore DPA concentration was 2.1 pg/μm^3^, approximately half that of the control strains *D. reducens* and *B. subtilis*.

Thus, results obtained from wet heat testing of spores of various clostridial species suggest that DPA/Ca content in spores is correlated to their wet heat resistance level, particularly with respect to short exposure to the treatment. After longer exposure to wet heat (∼ 60 min), spores survival decrease dramatically in all strains, with the exception of *C. acetobutylicum* DSM 1731. In this strain, the survival of spores is identical after short wet heat treatment, thus, the survivors retain a relatively high ability to germinate and recover even after prolonged exposure to wet heat.

The relatively low DPA/Ca concentrations in *C. acetobutylicum* DSM 792 and *C. beijerinckii* DSM 791 spores could potentially be a consequence of environmental adaptation. However, the four strains *C. acetobutylicum* and *C. beijerinckii* strains considered in this study have essentially the same type of metabolism (fermentative and solventogenic), and all live in similar environments. Thus, there is little evidence that environmental conditions may lead to this difference.

Another hypothesis was that the strains that produce butanol rather than ethanol may produce more resistant spores. Indeed, *C. acetobutylicum* DSM 1731 and *C. beijerinckii* NCIMB 8052 are known to be some of the most efficient at producing butanol amongst the solventogenic Clostridia (Chua et al., 2013). Thus, we propose that difference in solvent yields amongst the strains considered may explain the heat resistance and Ca/DPA content discrepancies.

As discussed above, in all Clostridia belonging to Cluster I, EtfA, an electron transfer flavoprotein, is likely the physiological DPA synthase. Some species of this cluster actually contain more homologs of *etfA* in the genome, roles of which are not assigned up to date (Orsburn et al., 2010). The function of EtfA may also include the production of butanol (Boynton et al., 1996). Thus, solventogenic strains that produce abundant butanol (DSM 1731 and NCIMB 8052) may also produce abundant DPA through the activity of EtfA. This link between metabolism and sporulation could also explain the heterogeneity in average DPA content of spores amongst strains, which are otherwise closely related phylogenetically. We can speculate that the heterogeneity in DPA/Ca content of spores within a given spore population may be attributable to differences in expression level of *etf*A prior to sporulation.

*Bacillus subtilis*, the model organism for the study of physiological and morphological processes in spore formers, also possesses the *etf* locus (*etfA* and *etfB*) in the genome. The function of EtfA flavoprotein in *B. subtilis* resembles the one in solventogenic Clostridia in several aspects. Not only is this protein in *B. subtilis* also involved in fatty acid synthesis, but it also plays a role in de-acidification or the internal environment of the cell by oxidizing NADH and consuming excess protons, thus maintaining the proton motive force of cellular respiration and membrane potential of the cell (Barabesi et al., 2007; Marvasi et al., 2010). While in solventogenic Clostridia the *etf* locus is located in the same operon as solventogenic genes, in *B. subtilis* the *etf* locus is part of *lcfA* operon, which is involved in process of calcite formation during biofilm development (Barabesi et al., 2007). Although it may seem that the function of *etfA* differs greatly in the two systems, a connection can be found: the transfer of electrons between NADH/NAD+ and consequent regulation of complicated redox reactions. Products of the *etf* operon are thus involved in processes associated with pH homeostasis in the cell, such as solventogenesis in Clostridia, where repeated oxidizing of NADH to NAD+ favors formation of reaction intermediates which lead to the production of neutral solvents rather than volatile acids. Because of the buffering properties of calcium carbonate, the precipitation of calcite as a result of *B. subtilis* metabolism is also a way to maintain the intracellular and extracellular pH within the boundaries suitable for neutrophiles.

It is interesting to note that the only non-solventogenic *Clostridium* strain considered here, *C. collagenovorans*, exhibits the highest Ca and DPA concentrations and the highest spore survival rate to heat treatment among Clostridia.

Overall, this study shows that there is great variability in the calcium, DPA concentrations in *Clostridium* Cluster I spores and that those concentrations correlate well with spore heat resistance. In addition, heat resistance also appears to correlate with the type of metabolism supported by specific organisms. Solventogenic strains generally harbor less DPA/Ca than non-solventogenic strains and within solventogenic strains, the ones producing more butanol appear to harbor more Ca/DPA and hence present greater heat resistance. We propose that the link between DPA synthesis and butanol production is through the *etf*A gene that serves in both pathways. Additional works is required to pinpoint the mechanistic underpinnings of this finding.

## Author Contributions

JJ carried out the STXM work and initiated the project. ZC and CL carried out most of the experimental work. BB did the electron microscopy. IB and RB-L designed the project and RB-L, ZC, and IB wrote the manuscript.

## Conflict of Interest Statement

The authors declare that the research was conducted in the absence of any commercial or financial relationships that could be construed as a potential conflict of interest.

## References

[B1] Ammann A. B. Kölle L. Brandl H. (2011). Detection of bacterial endospores in soil by terbium fluorescence. *Int. J. Microbiol.* 2011:435281 10.1155/2011/435281 PMC3132637 21754939

[B2] Barabesi C. Galizzi A. Mastromei G. Rossi M. Tamburini E. Perito B. (2007). *Bacillus subtilis* gene cluster involved in calcium carbonate biomineralization. *J. Bacteriol.* 189 228–235. 10.1128/JB.01450-06 17085570 PMC1797216

[B3] Beaman T. C. Greenamyre J. T. Corner T. R. Pankratz H. S. Gerhardt P. (1982). Bacterial spore heat resistance correlated with water content, wet density, and protoplast/sporoplast volume ratio. *J. Bacteriol.* 150 870–877. 6802802 10.1128/jb.150.2.870-877.1982 PMC216440

[B4] Bernier-Latmani R. Veeramani H. Vecchia E. D. Junier P. Lezama-Pacheco J. S. Suvorova E. I. (2010). Non-uraninite products of microbial U(VI) reduction. *Environ. Sci. Technol.* 44 9456–9462. 10.1021/es101675a 21069950

[B5] Boynton Z. L. Bennet G. N. Rudolph F. B. (1996). Cloning, sequencing, and expression of clustered genes encoding beta-hydroxybutyryl-coenzyme A (CoA) dehydrogenase, crotonase, and butyryl-CoA dehydrogenase from *Clostridium acetobutylicum* ATCC 824. *J. Bacteriol.* 178 3015–3024. 8655474 10.1128/jb.178.11.3015-3024.1996 PMC178046

[B6] Brul S. van Beilen J. Caspers M. O’Brien A. de Koster C. Oomes S. (2011). Challenges and advances in systems biology analysis of Bacillus spore physiology; molecular differences between an extreme heat resistant spore forming *Bacillus subtilis* food isolate and a laboratory strain. *Food Microbiol.* 28 221–227. 10.1016/j.fm.2010.06.011 21315977

[B7] Chua T. K. Liang D.-W. Qi C. Yang K.-L. He J. (2013). Characterization of a butanol–acetone-producing *Clostridium* strain and identification of its solventogenic genes. *Bioresour. Technol.* 135 372–378. 10.1016/j.biortech.2012.08.085 23069614

[B8] Coleman W. H. Chen D. Li Y. Q. Cowan A. E. Setlow P. (2007). How moist heat kills spores of *Bacillus subtilis*. *J. Bacteriol.* 189 8458–8466. 10.1128/JB.01242-07 17890306 PMC2168948

[B9] Dalla Vecchia E. Visser M. Stams A. J. Bernier-Latmani R. (2014). Investigation of sporulation in the *Desulfotomaculum* genus: a genomic comparison with the genera *Bacillus* and *Clostridium*. *Environ. Microbiol. Rep.* 6 756–766. 10.1111/1758-2229.12200 25132579

[B10] Daniel R. A. Errington J. (1993). Cloning, DNA sequence, functional analysis and transcriptional regulation of the genes encoding dipicolinic acid synthetase required for sporulation in *Bacillus subtilis*. *J. Mol. Biol.* 232 468–483. 10.1006/jmbi.1993.1403 8345520

[B11] Dittmann C. Han H.-M. Grabenbauer M. Laue M. (2015). Dormant *Bacillus* spores protect their DNA in crystalline nucleoids against environmental stress. *J. Struct. Biol.* 191 156–164. 10.1016/j.jsb.2015.06.019 26094877

[B12] Douki T. Setlow B. Setlow P. (2005). Photosensitization of DNA by dipicolinic acid, a major component of spores of *Bacillus* species. *Photochem. Photobiol. Sci.* 4 591–597. 10.1039/b503771a 16052264

[B13] Dürre P. (2009). “Metabolic networks in*Clostridium acetobutylicum*: interaction of sporulation, solventogenesis and toxin formation,” in *Clostridia. Molecular Biology in the Post-genomic Era* eds Brüggemann H. Gottschalk G. (Norfolk: Caister Academic Press) 215–227.

[B14] Ghosh S. Setlow P. (2009). Isolation and characterization of superdormant spores of *Bacillus* species. *J. Bacteriol.* 191 1787–1797. 10.1128/JB.01668-08 19136594 PMC2648361

[B15] Ghosh S. Zhang P. Li Y. Setlow P. (2009). Superdormant spores of *Bacillus* species have elevated wet-heat resistance and temperature requirements for heat activation. *J. Bacteriol.* 191 5584–5591. 10.1128/JB.00736-09 19592590 PMC2737942

[B16] Gupta R. S. Gao B. (2009). Phylogenomic analyses of clostridia and identification of novel protein signatures that are specific to the genus *Clostridium sensu stricto* (cluster I). *Int. J. Syst. Evol. Microbiol.* 59 285–294. 10.1099/ijs.0.001792-0 19196767

[B17] Hitchcock A. P. (1997). *AXIS2000 Software, Last Version 2016*. Available at: http://unicorn.mcmaster.ca/aXis2000.html

[B18] Huang S. S. Chen D. Pelczar P. L. Vepachedu V. R. Setlow P. Yong-qing L. (2007). Levels of Ca2+-dipicolinic acid in individual *Bacillus* spores determined using microfluidic Raman tweezers. *J. Bacteriol.* 189 4681–4687. 10.1128/jb.00282-07 17468248 PMC1913426

[B19] Jacobsen C. Wirick S. Flynn G. Zimba C. (2000). Soft X-ray spectroscopy from image sequences with sub-100 nm spatial resolution. *J. Microsc.* 197 173–184. 10.1046/j.1365-2818.2000.00640.x 11543408

[B20] Jain M. K. Zeikus J. G. (1988). Taxonomic distinction of two new protein specific, hydrolytic anaerobes: isolation and characterization of *Clostridium proteolyticum* sp. nov. and *Clostridium collagenovorans* sp. nov. *Syst. Appl. Microbiol.* 10 134–141. 10.1016/S0723-2020(88)80027-4

[B21] Jain M. K. Zeikus J. G. (1989). Bioconversion of gelatin to methane by coculture of *Clostridium collagenovorans* and *Methanosarcina barkeri*. *Appl. Environ. Microbiol.* 55 366–371. 16347846 10.1128/aem.55.2.366-371.1989 PMC184116

[B22] Jamroskovic J. Shao P. Suvorova E. Barak I. Bernier-Latmani R. (2014). Combined scanning transmission X-ray and electron microscopy for the characterization of bacterial endospores. *FEMS Microbiol. Lett.* 358 188–193. 10.1111/1574-6968.12539 25048294

[B23] Keis S. Bennett C. F. Ward V. K. Jones D. T. (1995). Taxonomy and phylogeny of industrial solvent- producing clostridia. *Int. J. Syst. Bacteriol.* 45 693–705. 10.1099/00207713-45-4-693 7547288

[B24] Lee K. S. Bumbaca D. Kosman J. Setlow P. Jedrzejas M. J. (2008). Structure of a protein–DNA complex essential for DNA protection in spores of *Bacillus* species. *Proc. Natl. Acad. Sci. U.S.A.* 105 2806–2811. 10.1073/pnas.0708244105 18287075 PMC2268541

[B25] Li Y. Davis A. Korza G. Zhang P. Li Y. Q. Setlow B. (2012). Role of a SpoVA protein in dipicolinic acid uptake into developing spores of *Bacillus subtilis*. *J. Bacteriol.* 194 1875–1884. 10.1128/JB.00062-12 22328679 PMC3318455

[B26] Ludwig W. Schleifer K. Whitman B. W. (2009). “Taxonomic outline of the phylum Firmicutes,” in *Bergey’s Manual of Systematic Bacteriology* 2nd Edn Vol. 3 eds de Vos P. Garrity G. M. Jones D. Krieg N. R. Ludwig W. Rainey F. (New York, NY: Springer).

[B27] Maillard J.-Y. (2011). Innate resistance to sporicides and potential failure to decontaminate. *J. Hosp. Infect.* 77 204–209. 10.1016/j.jhin.2010.06.028 20850897

[B28] Marvasi M. Visscher P. T. Perito B. Mastromei G. Casillas-Martínez L. (2010). Physiological requirements for carbonate precipitation during biofilm development of *Bacillus subtilis* etfA mutant. *FEMS Microbiol. Ecol.* 71 341–350. 10.1111/j.1574-6941.2009.00805.x 20059546

[B29] O’Brien R. W. Morris J. G. (1971). Oxygen and the growth and metabolism of *Clostridium acetobutylicum*. *J. Gen. Microbiol.* 68 307–318. 10.1099/00221287-68-3-307 4332793

[B30] Onyenwoke R. U. Brill J. A. Farahi K. Wiegel J. (2004). Sporulation genes in members of the low G+C Gram-typepositive phylogenetic branch (Firmicutes). *Arch. Microbiol.* 182 182–192. 10.1007/s00203-004-0696-y 15340788

[B31] Orsburn B. C. Melville S. B. Popham D. L. (2008). Factors contributing to heat resistance of *Clostridium perfringens* endospores. *Appl. Environ. Microbiol.* 74 3328–3335. 18378644 10.1128/AEM.02629-07 PMC2423036

[B32] Orsburn B. C. Melville S. B. Popham D. L. (2010). EtfA catalyses the formation of dipicolinic acid in *Clostridium perfringens*. *Mol. Microbiol.* 75 178–186. 10.1111/j.1365-2958.2009.06975.x 19968785

[B33] Paidhungat M. Setlow B. Driks A. Setlow P. (2000). Characterization of spores of *Bacillus subtilis* which lack dipicolinic acid. *J. Bacteriol.* 182 5505–5512. 10.1128/JB.182.19.5505-5512.2000 10986255 PMC110995

[B34] Paredes-Sabja D. Setlow P. Sarker M. R. (2011). Germination of spores of *Bacillales* and *Clostridiales* species: mechanisms and proteins involved. *Trends Microbiol.* 19 85–94. 10.1016/j.tim.2010.10.004 21112786

[B35] Paredes-Sabja D. Shen A. Sorg J. A. (2014). *Clostridium difficile* spore biology: sporulation, germination, and spore structural proteins. *Trends Microbiol.* 22 406–416. 10.1016/j.tim.2014.04.003 24814671 PMC4098856

[B36] Raju D. Walters M. Setlow P. Sarker M. R. (2006). Investigating the role of small, acid-soluble spore proteins (SASPs) in the resistance of *Clostridium perfringens* spores to heat. *BMC Microbiol.* 6:50 10.1186/1471-2180-6-50 PMC1501028 16759397

[B37] Ravagnani A. Jennert K. C. B. Steiner E. Grunberg R. Jefferies J. R. Wilkinson S. R. (2000). Spo0A directly controls the switch from acid to solvent production in solvent-forming Clostridia. *Mol. Microbiol.* 37 1172–1185. 10.1046/j.1365-2958.2000.02071.x 10972834

[B38] Rosen D. L. Sharpless C. McGown L. B. (1997). Bacterial endospore detection and determination by use of terbium dipicolinate photoluminescence. *Anal. Chem.* 69 1082–1085. 10.1021/ac960939w

[B39] Setlow P. (1995). Mechanisms for the prevention of damage to DNA in spores of *Bacillus* species. *Annu. Rev. Microbiol.* 49 29–54. 10.1146/annurev.mi.49.100195.000333 8561462

[B40] Setlow P. (2006). Spores of *Bacillus subtilis*: their resistance to and killing by radiation, heat and chemicals. *J. Appl. Microbiol.* 101 514–525. 10.1111/j.1365-2672.2005.02736.x 16907802

[B41] Setlow P. (2013). Summer meeting 2013 – when the sleepers wake: the germination of spores of *Bacillus* species. *J. Appl. Microbiol.* 115 1251–1268. 10.1111/jam.12343 24102780

[B42] Setlow P. (2014). Spore resistance properties. *Microbiol. Spectr.* 2:TBS-0003-2012 10.1128/microbiolspec.TBS-0003-2012 26104355

[B43] Sutton S. (2010). The most probable number method and its uses in enumeration, qualification, and validation. *J. Valid Technol.* 16 35–38.

[B44] Tovar-Rojo F. Chander M. Setlow B. Setlow P. (2002). The products of the spoVA operon are involved in dipicolinic acid uptake into developing spores of *Bacillus subtilis*. *J. Bacteriol.* 184 584–587. 10.1128/JB.184.2.584-587.2002 11751839 PMC139579

[B45] Wang Y. Li X. Blaschek H. P. (2013). Effects of supplementary butyrate on butanol production and the metabolic switch in *Clostridium beijerinckii* NCIMB 8052: genome-wide transcriptional analysis with RNA-Seq. *Biotechnol. Biofuels* 6:138 10.1186/1754-6834-6-138 PMC3849199 24229082

[B46] Xiao Y. Francke C. Abee T. Wells-Bennik M. H. (2011). Clostridial spore germination versus bacilli: genome mining and current insights. *Food Microbiol.* 28 266–274. 10.1016/j.fm.2010.03.016 21315983

[B47] Youngman P. Perkins J. B. Losick R. (1984). Construction of a cloning site near one end of Tn917 into which foreign DNA may be inserted without affecting transposition in *Bacillus subtilis* or expression of the transposon-borne erm gene. *Plasmid* 12 1–9. 10.1016/0147-619X(84)90061-1 6093169

[B48] Zhang P. Kong L. Setlow P. Li Y. Q. (2010). Characterization of wet heat inactivation of single spores of *Bacillus* species by dual-trap Raman spectroscopy and elastic light scattering. *Appl. Environ. Microbiol.* 76 1796–1805. 10.1128/AEM.02851-09 20097820 PMC2837993

[B49] Zhang P. Kong L. Wang G. Setlow P. Li Y.-Q. (2011). Monitoring the wet-heat inactivation dynamics of single spores of *Bacillus* species using Raman tweezers, differential interference contrast and nucleic acid dye fluorescence microscopy. *Appl. Environ. Microbiol.* 77 4754–4769. 10.1128/AEM.00194-11 21602365 PMC3147409

